# Baseline characteristics and hospital mortality in the Acute Heart Failure Database (AHEAD) Main registry

**DOI:** 10.1186/cc10584

**Published:** 2011-12-07

**Authors:** Jindrich Spinar, Jiri Parenica, Jiri Vitovec, Petr Widimsky, Ales Linhart, Marian Fedorco, Filip Malek, Cestmír Cihalik, Lenka Spinarová, Roman Miklik, Marian Felsoci, Miroslav Bambuch, Ladislav Dusek, Jiri Jarkovsky

**Affiliations:** 1 Department of Internal Cardiology Medicine, University Hospital Brno, Jihlavska 20, Brno 625 00, Czech Republic; 2 Medical Faculty, Masaryk University, Kamenice 5, Brno 625 00, Czech Republic; 3 First Department of Internal Cardioangiology Medicine, University Hospital St.Anne's, Pekarska 53, Brno 656 91, Czech Republic; 4 Cardiocenter, University Hospital Kralovske Vinohrady and the Third Faculty of Medicine, Charles University, Srobarova 50, Prague 100 34, Czech Republic; 5 2nd Department of Internal Cardiovascular Medicine, First Medical Faculty, Charles University in Prague, General University Hospital in Prague, U Nemocnice 2, Prague 128 08, Czech Republic; 6 Internal Cardiology Department, University Hospital Olomouc, I.P. Pavlova 6, Olomouc 779 00, Czech Republic; 7 Department of Cardiology, Na Homolce Hospital, Roentgenova 2/37, Praha 150 30, Czech Republic; 8 Cardiovascular Department, T. Bata Regional Hospital, Havlickovo Nabrezi 600, Zlin 760 01, Czech Republic; 9 Institute of Biostatistics and Analyses, Faculty of Medicine, Masaryk University, Kamenice 126/3, 625 00 Brno, Czech Republic

**Keywords:** acute heart failure, AHEAD, in-hospital mortality, prognosis

## Abstract

**Introduction:**

The prognosis of patients hospitalized with acute heart failure (AHF) is poor and risk stratification may help clinicians guide care. The objectives of the Acute Heart Failure Database (AHEAD) registry are to assess patient characteristics, etiology, treatment and outcome of AHF.

**Methods:**

The AHEAD main registry includes patients hospitalized for AHF in seven centers with a Catheterization Laboratory Service in the Czech Republic. The data were collected from September 2006 to October 2009. The inclusion criteria for the database adhere to the European guidelines for AHF (2005) and patients were systematically classified according to the basic syndromes, type and etiology of AHF.

**Results:**

Of 4,153 patients, 12.7% died during hospitalization. The median length of hospitalization was 7.1 days. Mean age of patients was 71.5 ± 12.4 years; men were younger (68.6 ± 12.4 years) compared to women (75.5 ± 11.5 years) (*P *< 0.001). *De-novo *heart failure was seen in 58.3% of the patients. According to the classification of heart failure syndromes, acute decompensated heart failure (ADHF) was reported in 55.3%, hypertensive AHF in 4.4%, pulmonary edema in 18.4%, cardiogenic shock in 14.7%, high output failure in 3.3%, and right heart failure in 3.8%. The mortality of cardiogenic shock was 62.7%, of right AHF 16.7%, of pulmonary edema 7.1%, of high output HF 6.1%, whereas the mortality of hypertensive AHF or ADHF was < 2.5%. According to multivariate analyses, low systolic blood pressure, low cholesterol level, hyponatremia, hyperkalemia, the use of inotropic agents and norepinephrine were predictive parameters for in-hospital mortality in patients without cardiogenic shock. Severe left ventricular dysfunction and renal insufficiency were predictive parameters for mortality in patients with cardiogenic shock. Invasive ventilation and age over 70 years were the most important predictive factors for mortality in both genders with or without cardiogenic shock.

**Conclusions:**

The AHEAD Main registry provides up-to-date information on the etiology, treatment and hospital outcomes of patients hospitalized with AHF. The results highlight the highest risk patients.

## Background

Acute heart failure (AHF) is a major and rapidly growing problem responsible for several million hospitalizations worldwide [1,2]. Heart failure (HF) causes considerable morbidity and mortality, and produces a tremendous burden on health economics worldwide.

The European Society of Cardiology defines AHF as the rapid onset of symptoms and signs secondary to abnormal cardiac function [3]. The clinical classification of patients with AHF continues to evolve, and reflects ongoing changes in the understanding of the pathophysiology of the syndrome [3-5]. AHF outcomes remain poor. Prevalence of in-hospital mortality as high as 10% and prevalence of re-hospitalization >50% within 1 year have been reported [6,7]. In the prospective cohort of hospitalized patients with AHF (ADHERE), in-hospital mortality was 4% [8]; the Second EuroHeart Failure Survey (EHFS II) had an in-hospital mortality of 6.7% [1].

Despite the magnitude of the burden of AHF and the intense interest in this dire problem, effective new therapies capable of reducing the prevalence of early mortality or re-hospitalization have not been developed over the past decade [7]. The etiology of AHF is mainly ischemic heart disease (IHD) [9]. Invasive methods in cardiology have significantly expanded in recent years.

The aim of this work is to describe a large population of patients hospitalized for syndromes of AHF, their in-patient therapy and mortality and to assess major risk factors of adverse short term prognosis in terms of frequently used invasive and therapeutic methods. The patients with AHF were systematically sorted according to AHF guidelines [3].

## Materials and methods

### Study populations

The Acute Heart Failure Database (AHEAD) registry consists of two independent parts. The AHEAD main registry includes consecutive patients in seven centers with a 24-hour Catheterization Laboratory service and centralized care for patients with acute coronary syndromes (ACS) from a region of about three million inhabitants. The AHEAD network also includes five regional hospitals without a Catheterization Laboratory service. The present work includes only patients from the AHEAD main registry.

The inclusion criteria for the database adhere to the European guidelines for AHF. Hence, there must be the signs and symptoms of HF, confirmed left-ventricular dysfunction (systolic or diastolic) and/or positive response to therapy [3]. The decision on inclusion in the registry and filling the database were done by responsible cardiologists. There was no exclusion criterion. Patients were systematically classified according to the type of AHF (*de novo *or acute decompensation of chronic heart failure), etiology of AHF (acute coronary syndrome, chronic coronary artery disease, valvular disease, arrhythmia, hypertensive crisis, and so on) and six basic syndromes of AHF defined according to ESC guidelines [3]: 1) acute decompensated heart failure (ADHF - with signs and symptoms of AHF, which are mild and do not fulfill criteria for cardiogenic shock, pulmonary edema or hypertensive crisis); 2) hypertensive AHF (symptoms of AHF are accompanied by high blood pressure on admission and relatively preserved left ventricular function with a chest radiograph compatible with acute pulmonary edema); 3) pulmonary edema (accompanied by severe respiratory distress, with crackles over the lungs and orthopnea with O_2 _saturation usually <90% prior treatment); 4) cardiogenic shock (defined as evidence of tissue hypoperfusion induced by heart failure after correction of preload, mostly with systolic BP <90 mmHg ongoing for at least 30 minutes); 5) high output failure (characterized by high cardiac output, usually with high heart rate often caused by arrhythmias, thyrotoxicosis crisis and anemia); and 6) right heart failure (characterized by low output syndrome with increased jugular venous pressure, increased liver size and hypotension).

Atrial fibrillation was defined as arrhythmia at admission and it was not distinguished whether it was a type of paroxysmal, persistent or permanent.

The AHEAD main registry included 4,153 patients hospitalized at seven Cardiology Departments with Catheterization Laboratory facilities in four cities. Data were collected prospectively from September 2006 until October 2009 using a database accessible via the Internet website http://www.ahead.registry.cz, and were evaluated continuously (including in-hospital mortality). The long-term mortality was followed using a centralized database of the Ministry of Health of the Czech Republic and will be published separately. Written informed consent was obtained from all subjects. The study protocol complied with the Declaration of Helsinki, and was approved by the local Ethics Committee of the Faculty Hospital Brno (Brno, Czech Republic).

### Statistical analysis

Statistical analyses were performed by the Institute of Biostatistics and Analyses of Masaryk University (Brno, Czech Republic). Standard summary statistics were used to describe primary data, absolute and relative frequencies, median, the 5^th ^to 95^th ^percentile range, arithmetic means and standard deviation. The statistical significance of differences between groups of patients in continuous parameters was tested using the Mann-Whitney U test. The Fisher exact test and maximum likelihood c^2 ^test were applied for the analyses of differences in some of the categories.

The relationship between hospital mortality and its potential predictors was analyzed by univariate logistic regression and described by odds ratios, their 95% confidence intervals (CI) and corresponding statistical significance. Multivariate logistic regression combining expert selection of predictors with a forward stepwise selection algorithm was used for the definition of the multivariate model for in-hospital mortality.

A level of α = 0.05 was used as the boundary for statistical significance in all analyses. Due to the large sample size, all statistical results were interpreted with respect to their clinical significance. Statistical analyses were undertaken using the SPSS 18.0.3 statistical package (SPSS, Chicago, IL, USA).

## Results

### Baseline characteristics

Of 4,153 patients, 526 (12.7%) died during hospitalization. The median length of hospitalization was 7.1 days (5.5 days for those patients who died and 9.7 days for those who were discharged home) and was identical for men and women.

**Figure 1:**
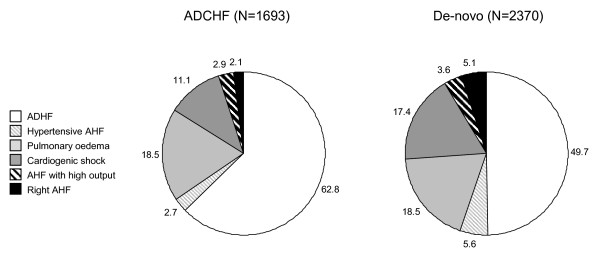
**Syndromes according to acute decompensation of chronic heart failure and *de-novo *acute heart failure**. ADCHF - Acute decompensation of chronic heart failure, ADHF - Acute decompensated heart failure, AHF - Acute heart failure.

The baseline characteristics according to the syndromes of AHF of patients enrolled in the AHEAD registry are shown in Table 1. The difference among individual syndromes in patients with *de-novo *and acute decompensation of chronic heart failure is shown in Figure 1. The baseline laboratory parameters are shown in Table 2. At admission hyponatremia (<130 mmol/L) was found in 5.0% of patients, hyperkalemia (>5.5 mmol/l) in 3.9% of patients and anemia (<120 g/L women, 130 g/L men) in 35.1% of patients. Medications being taken on admission and in surviving patients on discharge from the hospital are shown in Table 3. When comparing the medication of patients with pre-existing chronic heart failure on admission and at discharge, we observed a significant increase of ACEI (from 58.0% to 67.1%), beta-blockers (from 64.1% to 78.2%), diuretics (from 76.0% to 94.9%), spironolactone (from 41.2% to 71.7%), antiarrhytmics (from 17.5% to 22.0%) and digoxin (from 25.5% to 28.6%) during hospitalization.

**Table 1:** Baseline characteristics of patients according to syndromes of acute heart failure.

Patients characteristics	**Total**^1 ^**(N = 4,153)**	ADHF (N = 2,241)	Hypertensive AHF (N = 179)	Pulmonary edema (N = 748)	Cardiogenic shock (N = 600)	AHF with high output (N = 132)	Right AHF (N = 156)	***P***^2^
Female	1,761 (42.4%)	905 (40.4%)	117 (65.4%)	330 (44.1%)	234 (39.0%)	70 (53.0%)	68 (43.6%)	**<0.001**
Age	73.8 (49.3; 87.9)	73.8 (48.5; 87.6)	74.8 (49.3; 88.7)	73.8 (53.8; 88.3)	74.3 (50.3; 87.9)	76.2 (51.0; 91.1)	65.8 (35.3; 84.9)	**<0.001**
>70 years	2,492 (60.0%)	1,342 (59.9%)	110 (61.5%)	468 (62.6%)	360 (60.0%)	93 (70.5%)	64 (41.0%)	**<0.001**
*De-novo *HF	2,421 (58.3%)	1,179 (52.6%)	133 (74.3%)	437 (58.4%)	412 (68.7%)	84 (63.6%)	121 (77.6%)	**<0.001**
ACS at admission	1,503 (36.2%)	742 (33.1%)	0 (0.0%)	305 (40.8%)	368 (61.3%)	10 (7.6%)	40 (25.6%)	**<0.001**
NYHA III+IV	1,794 (43.2%)	1,094 (48.8%)	61 (34.0%)	323 (43.2%)	163 (27.2%)	49 (37.2%)	46 (29.5%)	**<0.001**
Insignificant CAD	577 (13.9%)	354 (15.8%)	40 (22.3%)	93 (12.4%)	33 (5.5%)	18 (13.6%)	24 (15.4%)	**<0.001**
Significant CAD	2,118 (51.0%)	1,154 (51.5%)	29 (16.2%)	411 (55.0%)	390 (65.0%)	27 (20.6%)	50 (32.1%)	**<0.001**
CAD - unknown	1,458 (35.1%)	733 (32.7%)	110 (61.5%)	244 (32.6%)	177 (29.5%)	87 (65.8%)	82 (52.5%)	**<0.001**
Chronic hypertension	3,036 (73.1%)	1,578 (70.4%)	169 (94.3%)	604 (80.8%)	433 (72.1%)	90 (68.0%)	95 (60.7%)	**<0.001**
Diabetes mellitus	1,769 (42.6%)	921 (41.1%)	77 (43.1%)	381 (51.0%)	264 (44.0%)	48 (36.2%)	38 (24.5%)	**<0.001**
Previous MI	1,333 (32.1%)	708 (31.6%)	47 (26.4%)	275 (36.8%)	216 (36.0%)	31 (23.6%)	18 (11.3%)	**<0.001**
Previous PCI or CABG	1,225 (29.5%)	650 (29.0%)	6 (3.4%)	215 (28.7%)	271 (45.2%)	12 (9.1%)	39 (25.0%)	**<0.001**
PM/ICD/CRT	507 (12.2%)	325 (14.5%)	15 (8.4%)	87 (11.6%)	45 (7.5%)	13 (9.8%)	10 (6.4%)	**<0.001**
COPD	673 (16.2%)	374 (16.7%)	32 (17.8%)	123 (16.5%)	86 (14.4%)	27 (20.5%)	12 (8.0%)	**0.027**
Stroke or TIA in history	685 (16.5%)	359 (16.0%)	47 (26.4%)	135 (18.0%)	94 (15.7%)	18 (13.4%)	15 (9.9%)	**0.002**
Atrial fibrillation	1,101 (26.5%)	634 (28.3%)	34 (19.0%)	160 (21.4%)	118 (19.7%)	97 (73.5%)	30 (19.2%)	**<0.001**
Coronary angiography	727 (17.5%)	441 (19.7%)	21 (11.7%)	138 (18.5%)	70 (11.6%)	16 (12.1%)	19 (12.3%)	**<0.001**
Systolic BP	135 (80; 200)	136 (95; 195)	198 (140; 260)	145 (95; 218)	110 (55; 170)	140 (90; 180)	110 (60; 160)	**<0.001**
Systolic BP ≤100	648 (15.6%)	224 (10.0%)	0 (0.0%)	65 (8.7%)	266 (44.4%)	20 (15.2%)	56 (36.1%)	**<0.001**
Diastolic BP	80 (50; 110)	80 (60; 110)	100 (70; 150)	80 (60; 120)	65 (30; 95)	80 (58; 112)	70 (34; 100)	**<0.001**
Heart rate	90 (54; 142)	85 (52; 140)	93 (55; 140)	98 (63; 141)	90 (45; 136)	130 (70; 170)	90 (49; 146)	**<0.001**
Ejection fraction (%)	37 (16; 65)	36 (15; 65)	55 (30; 70)	35 (18; 60)	30 (12; 60)	52 (25; 70)	57 (25; 74)	**<0.001**
Ejection fraction ≤30%	1,574 (37.9%)	858 (38.3%)	10 (5.6%)	320 (42.8%)	316 (52.6%)	13 (10.1%)	19 (12.2%)	**<0.001**

^1^Syndromes are not known for 97 patients. ^2^Overall statistical significance of differences among syndromes is based on Kruskal-Wallis test for continuous variables and ML chi-square test for categorical variables. ACS, acute coronary syndrome; ADHF, acute decompensated heart failure; BP, blood pressure; CABG, coronary artery bypass graft; CAD, coronary artery disease; COPD, chronic obstructive pulmonary disease; CRT, cardiac resynchronisation therapy; ICD, implantable cardiac defibrillator; MI, myocardial infarction; PCI, percutaneous coronary intervention; PM, pacemaker; TIA, transient ischemic attack.

**Table 2:** Laboratory description of patients stratified according to gender.

Patient characteristic	Total (N = 4,153)	Men (N = 2,397)	Women (N = 1,756)	***P***^2^
Creatinine at admission (μmol/l)	109 (68; 241)	116 (75; 252)	100 (62; 220)	**<0.001**
Creatinine max. (μmol/l)	125 (75; 353)	130 (81; 362)	117 (70; 333)	**<0.001**
Na^+ ^(mmol/l)	139 (130; 144)	138 (130; 144)	139 (130; 145)	**0.003**
K^+ ^(mmol/l)	4.1 (3.2; 5.4)	4.2 (3.3; 5.4)	4.1 (3.1; 5.4)	**0.002**
Glycemia (mmol/l)	8.0 (4.8; 19.6)	7.8 (4.8; 18.5)	8.4 (4.9; 20.6)	**<0.001**
Hemoglobin (g/l)	132 (96; 162)	137 (98; 165)	126 (94; 153)	**<0.001**
NT-proBNP at entry^1 ^(pg/ml)	5,294 (285; 30 000)	5,873 (285; 30 000)	4,788 (329; 30 000)	0.378
BNP at entry^1 ^(pg/ml)	767 (38; 3 414)	750 (43; 3 712)	809 (18; 3 272)	0.911
Cholesterol (mmol/l)	4.4 (2.5; 6.8)	4.2 (2.4; 6.7)	4.6 (2.7; 7.0)	**<0.001**
Uric acid (μmol/l)	413 (218; 691)	432 (224; 700)	385 (203; 672)	**<0.001**

^1^Data available from fewer than 50% of patients. ^2^Statistical significance of differences between groups of patients is based on Mann-Whitney U.test. BNP, B-type natriuretic peptide; NT-proBNP, N-terminal pro-B-type natriuretic peptide.

**Table 3:** Pharmacotherapy of patients according to syndromes of acute heart failure.

**Medication on admission**^1^	Total (N = 4,153)	ADHF (N = 2,241)	Hypertensive AHF (N = 179)	Pulmonary edema (N = 748)	Cardiogenic shock (N = 600)	AHF with high output (N = 132)	Right AHF (N = 156)	***P***^3^
Antiplatelets	1,865 (44.9%)	988 (44.1%)	84 (47.1%)	406 (54.3%)	259 (43.1%)	48 (36.0%)	35 (22.2%)	**<0.001**
Anticoagulants	739 (17.8%)	459 (20.5%)	24 (13.4%)	99 (13.3%)	80 (13.3%)	26 (20.0%)	26 (16.7%)	**<0.001**
ACE inhibitors	1,973 (47.5%)	1,069 (47.7%)	94 (52.3%)	383 (51.2%)	262 (43.6%)	55 (41.6%)	62 (39.6%)	**0.016**
AT2	511 (12.3%)	273 (12.2%)	28 (15.7%)	95 (12.7%)	62 (10.4%)	21 (16.0%)	18 (11.8%)	0.414
Beta-blockers	2,118 (51.0%)	1,185 (52.9%)	100 (55.8%)	399 (53.4%)	263 (43.8%)	58 (44.0%)	54 (34.7%)	**<0.001**
Calcium antagonists	968 (23.3%)	459 (20.5%)	54 (30.2%)	222 (29.7%)	147 (24.5%)	32 (24.0%)	37 (23.6%)	**<0.001**
Diuretics	2,284 (55.0%)	1,307 (58.3%)	85 (47.7%)	408 (54.5%)	277 (46.2%)	70 (52.8%)	71 (45.8%)	**<0.001**
Spironolactone	951 (22.9%)	594 (26.5%)	22 (12.2%)	153 (20.4%)	108 (18.0%)	21 (16.0%)	25 (16.0%)	**<0.001**
Statins	1,325 (31.9%)	706 (31.5%)	53 (29.7%)	296 (39.6%)	172 (28.6%)	35 (26.4%)	28 (18.1%)	**<0.001**
Other antiarrhytmics	482 (11.6%)	289 (12.9%)	21 (11.6%)	78 (10.4%)	58 (9.6%)	18 (13.6%)	7 (4.2%)	**0.005**
Digoxin	702 (16.9%)	410 (18.3%)	32 (18.0%)	99 (13.3%)	93 (15.5%)	26 (20.0%)	19 (12.0%)	**0.016**
Nitrates	802 (19.3%)	412 (18.4%)	30 (16.9%)	188 (25.2%)	131 (21.9%)	19 (14.4%)	8 (4.9%)	**<0.001**

^2^**Medication at discharge**	**Total (N = 3,627)**	**ADHF (N = 2,184)**	**Hypertensive AHF (N = 175)**	**Pulmonary edema (N = 695)**	**Cardiogenic shock (N = 224)**	**AHF with high output (N = 124)**	**Right AHF (N = 130)**	

Antiplatelets	2,441 (67.3%)	1,474 (67.5%)	122 (69.5%)	511 (73.5%)	159 (71.2%)	62 (50.0%)	48 (36.9%)	**<0.001**
Anticoagulants	1,066 (29.4%)	612 (28.0%)	39 (22.4%)	170 (24.5%)	86 (38.5%)	53 (42.7%)	76 (58.5%)	**<0.001**
ACE inhibitors	2,514 (69.3%)	1,570 (71.9%)	125 (71.3%)	499 (71.8%)	120 (53.5%)	72 (58.1%)	66 (50.8%)	**<0.001**
AT2	370 (10.2%)	229 (10.5%)	33 (19.0%)	63 (9.1%)	9 (4.0%)	15 (12.1%)	11 (8.5%)	**<0.001**
Beta-blockers	2,782 (76.7%)	1,743 (79.8%)	135 (77.0%)	531 (76.4%)	139 (61.9%)	92 (74.2%)	71 (54.6%)	**<0.001**
Calcium antagonists	638 (17.6%)	341 (15.6%)	89 (51.1%)	138 (19.9%)	12 (5.3%)	21 (16.9%)	21 (16.2%)	**<0.001**
Diuretics	3,032 (83.6%)	1,850 (84.7%)	155 (88.5%)	626 (90.1%)	170 (75.7%)	95 (76.6%)	59 (45.4%)	**<0.001**
Spironolactone	2,060 (56.8%)	1,321 (60.5%)	63 (36.2%)	444 (63.9%)	87 (38.9%)	53 (42.7%)	32 (24.6%)	**<0.001**
Statins	2,093 (57.7%)	1,278 (58.5%)	86 (49.4%)	455 (65.5%)	119 (53.1%)	45 (36.3%)	54 (41.5%)	**<0.001**
Other antiarrhytmics	613 (16.9%)	360 (16.5%)	26 (14.9%)	112 (16.1%)	39 (17.3%)	52 (41.9%)	8 (6.2%)	**<0.001**
Digoxin	707 (19.5%)	454 (20.8%)	24 (13.8%)	105 (15.1%)	36 (15.9%)	53 (42.7%)	13 (10.1%)	**<0.001**
Nitrates	479 (13.2%)	317 (14.5%)	17 (9.8%)	106 (15.3%)	11 (4.9%)	11 (8.9%)	2 (1.5%)	**<0.001**

^1^Syndromes are not known for 97 patients. ^2^Only patients surviving at discharge from hospital (N = 3627). ^3^Overall statistical significance of differences among syndromes is based on ML chi-square test. ACE, angiotensin-converting enzyme; AT2, antagonist for type 2 receptor for angiotensin II; Other antiarrhytmics, amiodarone, sotahexal, propafenone.

**Figure 2:**
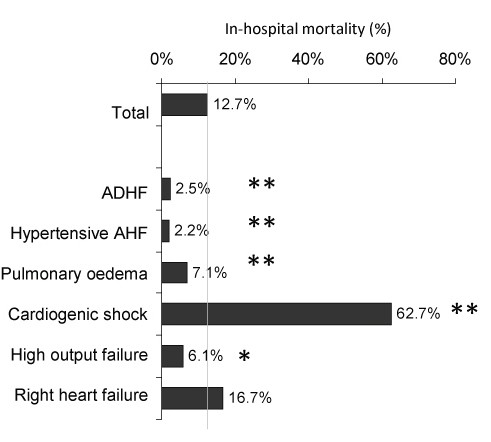
**In-hospital mortality according to syndromes of acute heart failure**. Statistical significance denoted as ** <0.001; *<0.050. ADHF - Acute decompensated heart failure, AHF - Acute heart failure.

**Figure 3:**
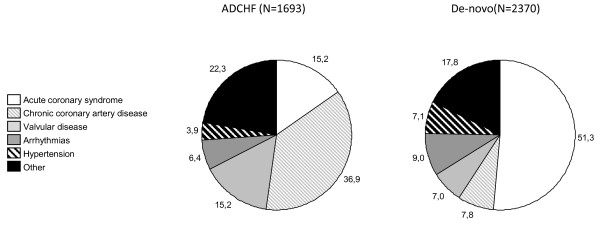
**The differences in etiologies between *de-novo *and acute decompensation of chronic heart failure**. ADCHF - Acute decompensation of chronic heart failure, De-novo - Acute heart failure de-novo.

Men were younger (68.6 ± 12.4 years) compared to women (75.5 ± 11.5 years) (*P *< 0.001) and in-hospital mortality was 13.0% for men and 12.2% for women (*P *= NS). Hypertensive AHF was a more frequent etiology in women than in men (8.5% versus 3.5%; *P *< 0.01). Men more frequently had ACS (37.1% versus 33.3%; *P *= 0.01), women had more comorbidities such as chronic hypertension, diabetes mellitus, rhythm disturbances or stroke. Patients with acute decompensation of chronic heart failure (N = 1,693, 42% of all patients) more frequently had comorbidities such as hypertension, diabetes mellitus and atrial fibrillation and their in-hospital mortality was 11.3% while in-hospital mortality of patients with *de-novo *HF was 14.0% (*P *< 0.05). Patients with acute coronary syndrome (ACS, N = 1,503, 36.2% of all patients) had lower in-hospital mortality than those without ACS (9.7% versus 18.1%; *P *< 0.01) and patients <70 years (N = 1,661, 40% of all patients) had lower in-hospital mortality than older patients (10.1% versus 14.4%; *P *< 0.01). The highest in-hospital mortality was in patients with cardiogenic shock and right acute heart failure; the lowest in-hospital mortality was in patients with hypertensive AHF (Figure 2). The most frequent etiologies of AHF were ACS (36.2%), chronic coronary artery disease (19.9%), valvular disease (10.4%), arrhythmias (7.9%) and hypertensive crisis (5.7%). The differences in etiologies between *de-novo *and acute decompensation of chronic heart failure are shown in Figure 3.

### Coronary angiography and percutaneous coronary intervention in patients with AHF

At admission 29.5% of patients had coronary revascularization by percutaneous coronary intervention or coronary artery bypass grafting in their medical history. During hospitalization coronary angiography was performed in 45.5% of patients, more often in younger patients <70 years old (69.8% versus 55.6%; *P *< 0.01) and in men (50.6% versus 41.0%; *P *< 0.001). Coronary angiography findings at the time of discharge were known in 62.6% of hospitalized patients in total. There were significant differences among syndromes of AHF (acute decompensated heart failure - 64.7%, hypertensive HF - 32.4%, pulmonary edema - 66.8%, cardiogenic shock - 70%, AHF with high output - 28.8%, and right AHF - 39.7%; *P *< 0.001). Percutaneous coronary intervention was performed in 25.3% of patients, in 80.4% of patients with AHF and myocardial infarction with ST segment elevations, in 38.5% of patients with AHF and myocardial infarction without ST elevations and in 4.9% of patients without ACS.

During the course of hospitalization, noradrenaline was used in 19.0%, adrenaline in 8.9%, dobutamine in 10.0%, dopamine in 8.7% and levosimendan in 3.7% of patients. The use of vasopressors and inotropes according to the clinical syndromes is shown in detail in Table 4. Administration of adrenaline was associated with in-hospital resuscitation (88.3%) and also with high in-hospital mortality (84.4%). Adrenaline only was used in 15% of all administrations; the most often combination was with noradrenaline in 34.7%. Although vasopressors were also administrated to patients with other syndromes then cardiogenic shock, these patients did not meet the criteria for cardiogenic shock according to the attending physicians. They had either no signs of tissue hypoperfusion and the treatment with vasopressors was only short-term with low doses or vasopressors were administered for another indication than cardiogenic shock, such as hemorrhagic shock, septic shock, hypovolemia, and so on. Hemodialysis was used in 2.4% of hospitalized patients, intra-aortic balloon contrapulsation (IABC) was used in 3.5% (N = 144) of all hospitalized patients (according to syndromes: in cardiogenic shock in 79.9% of patients; in pulmonary edema in 9.0% of patients; in acute decompensated heart failure in 11.9%of patients; according to etiology: in patients with ACS in 91.1%; in patients with valvular disease in 4.9%; in patients with chronic coronary artery disease in 4.0%). Pulmonary ventilation was used in 25.0% (non-invasive ventilation in 11.1%, invasive ventilation in 16.1%). There were 94 patients who needed both invasive and non-invasive ventilation mostly because of no clinical improvement after non-invasive ventilation. The use of ventilator support according to the clinical syndromes is shown in Table 5 and the in-hospital mortality according to the type of ventilatory support used is shown in Table 6. Patients who required invasive ventilation had higher hospital mortality (13.9% versus 52.8%; *P *< 0.001) which was determined above all by heart failure severity. Patients treated by non-invasive ventilation had milder forms of acute heart failure (acute decompensated heart failure 41.0%; pulmonary edema 35.6%; and cardiogenic shock 13.0%) in comparison with patients treated by invasive ventilation (acute decompensated heart failure 13.8%; pulmonary edema 20.0%; and cardiogenic shock 58.6%). We did not find significant differences in age, gender, ejection fraction or comorbidities (hypertension, diabetes mellitus, previous myocardial infarction, or chronic obstructive pulmonary disease (COPD)) between the two groups. Patients requiring invasive ventilation had slightly higher levels of creatinine at admission (median 109 versus 126 μmol/L; *P *< 0.001).

**Table 4:** Pharmacotherapy by vasopressors and inotropes according to the syndromes of acute heart failure.

	Total (N = 4,153)	ADHF (N = 2,241)	Hypertensive AHF (N = 179)	Pulmonary edema (N = 748)	Cardiogenic shock (N = 600)	AHF with high output (N = 132)	Right AHF (N = 156)	***P***^**1**^
Noradrenaline	770 (19.0%)	132 (5.9%)	5 (2.8%)	128 (17.2%)	438 (73.6%)	11 (8.3%)	52 (33.5%)	<0.001*
Adrenaline	360 (8.9%)	35 (1.6%)	1 (0.6%)	31 (4.2%)	268 (44.9%)	1 (0.8%)	21 (13.5%)	<0.001*
Dobutamine	407 (10.0%)	95 (4.3%)	1 (0.6%)	77 (10.3%)	215 (36.1%)	4 (3.0%)	13 (8.4%)	<0.001*
Dopamine	352 (8.7%)	108 (4.9%)	4 (2.2%)	51 (6.8%)	149 (25.0%)	8 (6.1%)	30 (19.4%)	<0.001*
Levosimendan	148 (3.6%)	43 (1.9%)	1 (0.6%)	31 (4.2%)	69 (11.6%)	-	3 (1.9%)	<0.001*

^1^Overall statistical significance of differences among syndromes is based on ML chi-square test

**Table 5:** Use of ventilation support according to the clinical syndromes.

	Total (N = 4,153)	ADHF (N = 2,241)	Hypertensive AHF (N = 179)	Pulmonary edema (N = 748)	Cardiogenic shock (N = 600)	AHF with high output (N = 132)	Right AHF (N = 156)	***P***^**1**^
No ventilatory support	3,116 (75.0%)	1,998 (89.2%)	143 (79.9%)	483 (64.6%)	160 (26.7%)	115 (87.1%)	121 (77.6%)	<0.001*
Only NIV	368 (8.9%)	151 (6.7%)	24 (13.4%)	131 (17.5%)	48 (8.0%)	3 (2.3%)	11 (7.1%)	
Only invasive ventilation	575 (13.8%)	83 (3.7%)	10 (5.6%)	111 (14.8%)	333 (55.5%)	14 (10.6%)	23 (14.7%)	
Both NIV and invasive ventilation	94 (2.3%)	9 (0.4%)	2 (1.1%)	23 (3.1%)	59 (9.8%)	-	1 (0.6%)	

^1^Overall statistical significance of differences among syndromes is based on ML chi-square test. ADHF - Acute decompensated heart failure, AHF - Acute heart failure, NIV - non-invasive ventilation.

**Table 6:** Hospital mortality according to the ventilatory support used and syndromes of acute heart failure.

	Total (N = 4,153)	ADHF (N = 2,241)	Hypertensive AHF (N = 179)	Pulmonary edema (N = 748)	Cardiogenic shock (N = 600)	AHF with high output (N = 132)	Right AHF (N = 156)
Total	N = 526 (12.7%)	N = 57 (2.5%)	N = 4 (2.2%)	N = 53 (7.1%)	N = 376 (62.7%)	N = 8 (6.1%)	N = 26 (16.7%)
No ventilatory support	122 (3.9%)	29 (1.5%)	1 (0.7%)	17 (3.5%)	64 (40.0%)	4 (3.5%)	5 (4.1%)
Only NIV	51 (13.9%)	2 (1.3%)	1 (4.2%)	12 (9.2%)	33 (68.8%)	-	3 (27.3%)
Only invasive ventilation	305 (53.0%)	24 (28.9%)	2 (20.0%)	19 (17.1%)	239 (71.8%)	4 (28.6%)	17 (73.9%)
NIV and invasive ventilation	48 (51.1%)	2 (22.2%)	-	5 (21.7%)	40 (67.8%)	-	1 (100.0%)
*P*^1^	<0.001*	<0.001*	0.044*	<0.001*	<0.001*	0.012*	<0.001*

^1^Statistical significance of differences in mortality among ventilaton categories is based on ML chi-square test.

Percentage in brackets indicates the number of deaths from the total number of patients with the clinical syndrome and ventilation mode from Table 5. ADHF - Acute decompensated heart failure, AHF - Acute heart failure, NIV - Non-invasive ventilation.

### Cardiogenic shock

In our study, 14.5% (N = 600) of patients were hospitalized with cardiogenic shock. In comparison with patients without cardiogenic shock, we did not find significant differences in age, gender, body mass index (BMI), diabetes mellitus, hypertension or COPD. *De-novo *acute heart failure was more frequent in patients with cardiogenic shock (68.3% versus 55.2% in patients without shock; *P *< 0.001) and acute coronary syndrome was the most widespread etiology of shock (61.3% versus 31.1% in patients without shock; *P *< 0.001). Patients with cardiogenic shock had higher blood glucose (10.8 mmol/L versus 7.7 mmol/L; *P *< 0.001), creatinine (129 μmol/L versus 107 μmol/L; *P *< 0.001) and lower blood pressure (BP) on admission (110/65 mmHg versus 140/80 mmHg; *P *< 0.001). Patients with shock needed more intense treatment: adrenaline was used in 44.9%, noradrenaline in 73.6%, dobutamine in 36.1% and dopamine in 25.0% of patients; 19.3% of patients received IABC (Table 4).

### Predictors of in-hospital mortality

The univariate and multivariate models of in-hospital mortality predictors are shown in Table 7. Due to the importance of cardiogenic shock for in-hospital mortality, the analysis was computed separately for patients with and without cardiogenic shock. Pulmonary ventilation and age were important prognostic parameters for patients with and without cardiogenic shock. Severe left ventricular systolic dysfunction and higher creatinine at admission were independent predictors of in-hospital mortality in patients with cardiogenic shock while parameters such as low systolic blood pressure, hyponatremia, low cholesterol and use of inotropes and noradrenaline were negative prognostic parameters only in patients without cardiogenic shock.

**Table 7:** The univariate and multivariate models of in hospital mortality predictors

	With cardiogenic shock (N = 600; in-hospital mortality = 62.7%)				Without cardiogenic shock (N = 3,553; in-hospital mortality = 4.2%)			
	Univariate (crude)		Multivariate adjusted		Univariate (crude)		Multivariate adjusted	
**Model parameter**	**OR (95% CI)**	***P***	**OR (95% CI)**	***P***	**OR (95% CI)**	***P***	**OR (95% CI)**	***P***

Age at hospital admission >70 years	1.9 (1.4-2.7)	**<0.001**	2.0 (1.3-3.0)	**0.001**	1.8 (1.3-2.6)	**0.001**	2.7 (1.7-4.1)	**<0.001**
BMI ≤28 (kg/m^2^)	0.7 (0.5-1.0)	**0.045**			1.0 (0.7-1.4)	0.947		
*De-novo *failure	0.6 (0.4-0.8)	**0.004**			1.3 (0.9-1.8)	0.167		
Diabetes mellitus	1.4 (1.0-2.0)	**0.035**			1.2 (0.9-1.7)	0.286		
DBP ≤60 (mmHg)	1.2 (0.9-1.7)	0.244			3.0 (2.1-4.3)	**<0.001**		
SBP ≤100 (mmHg)	1.0 (0.7-1.3)	0.710			3.6 (2.5-5.2)	**0.000**	1.6 (1.0-2.6)	**0.050**
Ejection fraction ≤30%	1.4 (1.0-2.0)	**0.050**	1.5 (1.0-2.2)	**0.034**	1.1 (0.8-1.7)	0.552		
Atrial fibrillation/flutter	1.3 (0.8-1.9)	0.284			1.0 (0.7-1.5)	0.807		
Haemoglobin ≤120 (g/l)	1.2 (0.8-1.7)	0.366			1.6 (1.1-2.3)	**0.010**		
Na^+ ^≤130 (mmol/l)	0.6 (0.3-1.0)	0.065			3.5 (2.1-5.9)	**<0.001**	2.4 (1.3-4.7)	**0.007**
Cholesterol ≤3 (mmol/l)	1.0 (0.5-1.7)	0.891			2.5 (1.6-3.9)	**<0.001**	2.4 (1.4-4.2)	**0.002**
K^+ ^>5.5 (mmol/l)	1.6 (0.8-3.1)	0.195			4.3 (2.5-7.6)	**<0.001**	1.9 (1.0-3.9)	**0.062**
Creatinine at admission >120 (μmol/l)	1.4 (1.0-2.0)	**0.030**	1.5 (1.0-2.2)	**0.048**	1.8 (1.3-2.5)	**0.001**		
Uric acid >500 (μmol/l)	1.2 (0.7-1.8)	0.542			1.3 (0.8-1.9)	0.299		
Glycaemia >10 (mmol/l)	0.8 (0.6-1.1)	0.129			1.5 (1.1-2.1)	**0.015**		
Invasive ventilation	2.8 (2.0-4.0)	**<0.001**	2.9 (1.9-4.3)	**<0.001**	15.3 (10.8-21.8)	**<0.001**	5.5 (3.4-8.8)	**<0.001**
Dobutamine, dopamine, levosimendan	0.8 (0.6-1.2)	0.269			5.9 (4.2-8.4)	**<0.001**	1.7 (1.1-2.6)	**0.030**
Noradrenaline	1.7 (1.2-2.5)	**0.003**			13.9 (9.8-19.6)	**<0.001**	3.9 (2.4-6.4)	**<0.001**

BMI, body mass index; CI, confidence interval; DBP, diastolic blood pressure; HR, heart rate; OR, odds ratio; SBP, systolic blood pressure.

## Discussion

The AHEAD main registry is one of the largest national observational prospective databases of AHF. The register was designed as multicenter and prospective with long-term mortality follow-up [10]. The data are stored in a format that allows the creation of a single aggregate dataset for research. The same data have been collected for all patients, the characteristics of the data were defined prior to its collection and the data were collected in a systematic and prospective manner. The completion of monitored data was 96%. The comparison of registries of AHF is difficult; therefore, we tried to compare our results with some of them only (ADHERE [8], ALARM-HF [11,12], EFICA [13], EHFS I [14,15], EHFS II [1], FINN-AKVA [16], OPTIMIZE-HF [9]). Table 8 demonstrates the basic characteristics of recent comparable registries based on the classifications of The European Society of Cardiology (ESC) guidelines on diagnosis and treatment of AHF 2005 [3].

**Table 8:** Comparison of basic characteristics of AHF registries - AHEAD, ALARM-HF, EHFS II and FINN-AKVA.

	AHEAD	ALARM-HF	EHFS II	FINN-AKVA
N	4,,153	4953	3,580	620
Age (mean)	71.5	66-70	69.9	75.1
Male (%)	57.6%	62.4%	61.3%	50.4
De-novo AHF	58.3%	36.2%	37.1%	49.0%

**Syndromes of AHF**				

ADHF	55.3%	36.8%	65.4%	63.5%
Hypertensive AHF	4.4%	7.4%	11.4%	3.1%
Pulmonary edema	18.4%	36.7%	16.2%	26.3%
Cardiogenic shock	14.7%	11.7%	3.9%	2.3%
AHF with high output	3.3%	1.1%	NA	NA
Right AHF	3.8%	4.5%	3.2%	4.8%

**Characteristics of population and hospital outcomes**				

ACS at admission	36.2%	36,8%	30.2%	31.9%
Chronic hypertension	73.1%	70.2%	62.5%	54.7%
Diabetes mellitus	43,0%	45.3%	32.8%	32.3%
Anaemia	35.1%	14.4%	14.7%	NA
PCI during hospitalization	25.3%	12.8%	8.4%	NA
EF LV < 30%	37.9%	26.0%	29.9%	16.2%
Hospital mortality	12.7%	12.0%	6.7%	7.1%
Follow-up	Yes	No	3 month	Yes

NA - data were not evaluated or data are not available. ACS - Acute coronary syndrome, ADHF - Acute decompensated heart failure, PCI - percutaneous coronary intervention, EF LV - Ejection fraction of left ventricle.

### Comparison of populations with AHF

Our study population size is comparable with some of the other registries [1,11]. The structure of the AHEAD main registry is closest to the structure of EHFS II [1], FIN-AKVA [16] and ALARM-HF [11]; the systematic stratification of patients with AHF was based on the guidelines of ESC [3] and the whole spectrum of patients with AHF was covered. The average age of patients with AHF is in the range of 69 to 75 years; women hospitalized for AHF were significantly older than the men. Patients with ACS comprise one third of all patients. A higher percentage of patients with myocardial infarction with ST elevation in the AHEAD main registry was caused by enrollment of patients in specialized cardiovascular centers with a higher frequency of patients with ACS. The prevalence of underlying disease in our study was comparable with that previously reported: chronic hypertension (73.1% versus 54.7% to72%), diabetes mellitus (42.6% versus 32.3% to 46.0%) and atrial fibrillation (26.5% versus 25.0% to 31.0%) [1,13,14,16]. Atrial fibrillation was present slightly more often in patients with acute decompensation of chronic heart failure (HF) (31.8%) than in *de-novo *HF (22.7%). Generally, we could conclude that a typical man with HF was younger with ACS whereas a typical woman was older than 70 years of age with a non-ischemic etiology of HF.

### In-hospital mortality

The overall hospital mortality of 12.7% was comparable with the mortality of ALARM-HF (11%) [11]. A lower hospital mortality was reported in FINN-AKVA (7.1%) [16], ADHERE (4.0%) [8,9], EHFS I (6.9%) [14,15], EHFS II (6.7%) [1] and OPTIMIZE-HF (3.8%) [17]. The main differences in mortality were based on different characteristics of the study populations. Cardiogenic shock was diagnosed in only 4% of the EHFS II population, in less than 1% of the EHFS I population, in 2.3% of the FIN-AKVA population, in 11.7% of the ALARM-HF population and in 28.5% of the EFICA population. In our study, 14.7% of the cases had cardiogenic shock. The reported mortality for patients with cardiogenic shock was 28.6% in the FIN-AKVA study (but only 14 patients had cardiogenic shock), 43% in ALARM-HF, 39.6% in EHFS II and 57.8% in EFICA. In our study, the in-hospital mortality of patients with cardiogenic shock was 62.7%. Lower mortality was seen in patients with *de-novo *HF (58.8% versus 71.7%; *P *< 0.01) and in patients under 70 years old (53.3% versus 68.9%; *P *< 0.01). The higher number of patients with cardiogenic shock in the AHEAD registry is determined by centralized care for patients with ACS and severe forms of heart failure in cardiovascular centers which were included in the AHEAD main registry. The patient's mortality is influenced by the severity of heart failure which could be expressed by the need for vasopressor administration. For example, in EHFS II with a mortality of 39.6%, noradrenaline was administered in 24% of patients with shock, while in the AHEAD registry (mortality 62.7%) the administration of noradrenaline was required in 73% of patients.

On the other hand, very low in-hospital mortality was recorded in patients with hypertensive acute heart failure (2.2%) or in patients with acute decompensated heart failure (2.5%). Patients enrolled in clinical trials are usually younger, male, have fewer comorbidities, are appropriately treated and have a better prognosis than 'real life' patients. Therefore, data from international and/or national registries reflect more precisely the reality and can provide important information. The same information can be found in the AHEAD main registry (even though more men than women were included in this registry).

### Coronary angiography and percutaneous coronary intervention in patients with AHF

Acute coronary syndrome and chronic ischemic heart disease together accounted for 56.1% of the etiologies of AHF in the AHEAD main registry. A total of 29.5% of patients with AHF had percutaneous coronary intervention (PCI) or coronary artery bypass grafting in their medical history. Recent results of coronary angiography were known in 17.5% of the admitted patients. Coronary angiography was performed in 45.5% of the hospitalized patients. At discharge coronary angiography findings were known in 62.6% of the patients, more often in patients with ACS (87.2% versus 47.1% in patients without ACS). During hospitalization PCI was performed mainly in patients with ACS; only 4.9% of patients without ACS were treated by PCI. In comparison angiography was done in 36.5% of the study population from EHFS II [1] and any revascularization (thrombolysis, PCI or CABG) was performed in 62% of the patients with myocardial infarction with ST elevation in the same study. The OPTIMIZE-HF registry evaluated 48,612 patients hospitalized for HF. In that registry coronary artery disease (CAD) was strongly associated with short- and long-term prognosis [17]. It is quite surprising that only 949 patients (<2%) underwent coronary revascularization during the index hospitalization in this survey.

### Predictors of in-hospital mortality

We divided patients into those with cardiogenic shock (N = 600) and very high mortality (62.7%) and those without cardiogenic shock (N = 3,553) with low mortality rate (4.2%). That was similar to the ADHERE registry [8,9] and even lower than in the ESHF II registry [1]. Using univariate logistic regression analyses we defined all parameters that were related to in-hospital mortality. Cardiopulmonary resuscitation and the use of adrenalin were excluded from the models. Patients with cardiogenic shock who were over 70 years old, with ejection fraction (EF) <30%, with renal insufficiency and treated with invasive pulmonary ventilation were at high risk of mortality. Age over 70 years, low systolic blood pressure, low cholesterol level, hyponatremia, hyperkalemia, the use of any inotropic agents and norepinephrine and the use of invasive pulmonary ventilation were independent predictive parameters for in-hospital mortality in patients without cardiogenic shock. In the OPTIMIZE HF registry [17], the strongest predictor of mortality in 48,612 patients were low systolic BP, hyponatremia, high levels of creatinine, and left-ventricular dysfunction [18].

### Treatment

According to our results, there were only 64% of patients who had been treated with beta-blockers and 58% of patients treated with ACEI in the group with pre-existing knowledge of heart failure on admission. These data are comparable with other registries (EHFS II, FIN-AKVA, OPTIMIZE-HF). In comparison with admission, at discharge there was a significant increase in all classes of drugs indicated for the treatment of heart failure (Table 3). The most common causes for not recommending the optimal medication at discharge were as follows: a tendency to hypotension at discharge, a tendency to bradycardia at discharge or instability of patients transferred to another department. Lack of the optimal medication at discharge could be a cause of recurrent acute decompensation but, at the same time, intolerance of these drugs, particularly the tendency to hypotension, is an adverse prognostic marker.

We found the total frequency of IABC use to be comparable with other registries (range of other registries 0.5% to 4.9%, AHEAD 3.5%) but the device was used less in patients in cardiogenic shock (range 22% to 40%, AHEAD 19.3%) [1,11,16,19,20]. The reason for less frequent use is not clear as IABC is commonly available in all hospitals with a Catheterization Laboratory without any restrictions on use. The use of IABC is recommended in the presence of hemodynamic impairment when low coronary perfusion is suspected (particularly with those in cardiogenic shock and with mechanical complications during ACS).

### Study limitations

The results presented here are only from hospitals with a Catheterization Laboratory service (AHEAD main). Results from regional hospitals participating in the AHEAD network are not included. This could have led to a higher contribution of patients with ACS and a high percentage of patients who had undergone coronary angiography and PCI during hospitalization. Despite recommendations at the beginning of the study, natriuretic peptides levels were determined in only half of the patients.

## Conclusion

The AHEAD main registry provides up-to-date information on the demographic characteristics and the underlying conditions of AHF patients as well as the etiology, investigation, treatment and prognosis of AHF in a country with centralized care for ACS and with a high percentage of patients who had received angiography and coronary revascularization. The AHEAD registry clearly demonstrates the gender differences of the patients admitted with AHF: women were older with higher SBP and more frequently preserved EF. The prognosis of those with cardiogenic shock was poor; the prognosis of patients with AHF without cardiogenic shock was similar to that observed in other reports. We defined the predictors of in-hospital mortality, since these parameters should alert the physician to patients at high risk of mortality.

## Key Messages

• The most frequent etiologies of acute heart failure in hospitalized patients were ACS (36.2%), chronic ischemic heart disease (19.9%), valvular disease (10.4%), arrhythmias (7.9%) and hypertensive crisis (5.7%).

• The overall in-hospital mortality was 12.7%. Patients with acute coronary syndrome had lower mortality than those without ACS (9.7% versus 18.1%). The highest mortality was in the patients with cardiogenic shock (62.7%) while there was a very low mortality in patients with acute decompensated heart failure (2.5%) and hypertensive acute heart failure (2.2%).

• We found a frequent use of invasive methods: during hospitalization coronary angiography was performed in 45.5% of patients, percutaneous coronary intervention in 25.3% and intra-aortic balloon contrapulsation was used in 19.3% of patients with cardiogenic shock.

• Age >70 years, ejection fraction of left ventricle ≤30% and mild renal insufficiency with creatinine at admission >120 μmol/l were adverse prognostic parameters in patients with cardiogenic shock.

• Age >70 years, systolic blood pressure ≤100 mmHg at admission, hyponatremia (Na^+ ^≤130 mmol/l), hypocholesterolemia (cholesterol ≤3 mmol/l), hyperkalemia (K^+ ^>5.5 mmol/l) and the use of invasive ventilation, inotropes and vasopressors were adverse prognostic parameters in patients without cardiogenic shock.

## List of abbreviation

ACE: angiotensin-converting enzyme; ACS: acute coronary syndrome; ADHF: acute decompensated heart failure; AHEAD: Acute Heart Failure Database; AHF: acute heart failure; AT2: antagonist for type 1 receptor for angiotensin II; BNP: B-type natriuretic peptide; BP: blood pressure; CABG: coronary artery bypass graft; CAD: coronary artery disease; COPD: chronic obstructive pulmonary disease; CRT: cardiac resynchronization therapy; DBP: diastolic blood pressure; EHFS II: Second EuroHeart Failure Survey; IABC: intra-aortic balloon contrapulsation; ICD: implantable cardiac defibrillator; IHD: ischemic heart disease; MI: myocardial infarction; NIV: non-invasive ventilation; NT-proBNP: N-terminal pro-B-type natriuretic peptide; PCI: percutaneous coronary intervention; PM: pacemaker; SBP: systolic blood pressure; TIA: transient ischemic attack.

## Competing interests

The authors declare that they have no competing interests.

## Authors' contributions

JS drafted the manuscript and participated in the study design, JP, JV, PW, AL, MF, FM, CC, LS, RM, MB and MF participated in the study design and helped to draft the manuscript. JJ and LD performed the statistical analysis and helped to draft the manuscript. All authors have read and approved the final manuscript.
